# Neuroendocrine Associations Underlying the Persistent Therapeutic Effects of Classic Serotonergic Psychedelics

**DOI:** 10.3389/fphar.2018.00177

**Published:** 2018-03-01

**Authors:** Emmanuelle A. D. Schindler, Ryan M. Wallace, Jordan A. Sloshower, Deepak C. D’Souza

**Affiliations:** ^1^Department of Neurology, Yale School of Medicine, New Haven, CT, United States; ^2^Department of Neurology, VA Connecticut Healthcare System, West Haven, CT, United States; ^3^Department of Psychiatry, Yale School of Medicine, New Haven, CT, United States; ^4^Department of Psychiatry, VA Connecticut Healthcare System, West Haven, CT, United States

**Keywords:** psychedelics, hallucinogens, neuroendocrine, circadian rhythm, cluster headache, depression, PTSD, substance use disorders

## Abstract

Recent reports on the effects of psychedelic-assisted therapies for mood disorders and addiction, as well as the effects of psychedelics in the treatment of cluster headache, have demonstrated promising therapeutic results. In addition, the beneficial effects appear to persist well after limited exposure to the drugs, making them particularly appealing as treatments for chronic neuropsychiatric and headache disorders. Understanding the basis of the long-lasting effects, however, will be critical for the continued use and development of this drug class. Several mechanisms, including biological and psychological ones, have been suggested to explain the long-lasting effects of psychedelics. Actions on the neuroendocrine system are some such mechanisms that warrant further investigation in the study of persisting psychedelic effects. In this report, we review certain structural and functional neuroendocrinological pathologies associated with neuropsychiatric disorders and cluster headache. We then review the effects that psychedelic drugs have on those systems and provide preliminary support for potential long-term effects. The circadian biology of cluster headache is of particular relevance in this area. We also discuss methodologic considerations for future investigations of neuroendocrine system involvement in the therapeutic benefits of psychedelic drugs.

## Introduction

In past decades, there has been a resurgence of interest in the therapeutic potential of classic serotonergic psychedelic drugs, such as psilocybin, lysergic acid diethylamide (LSD), and *N,N*-dimethyltryptamine (DMT), all compounds that bind and activate serotonin (5-hydoxytryptamine, 5-HT) 2A receptors. Psilocybin has been reported to treat depression and anxiety in cancer patients (Grob et al., 2011; Gasser et al., 2015; Griffiths et al., 2016), obsessive-compulsive symptoms (Moreno et al., 2006), and alcohol and tobacco addictions (Garcia-Romeu et al., 2014; Bogenschutz et al., 2015; Johnson et al., 2017a,b), as well as enhance attitude, mood, and behavior (Griffiths et al., 2008, 2011, 2016). In early studies, LSD has been shown to be effective in the treatment of alcoholism (Krebs and Johansen, 2012) and it improved affect and sleep while reducing pain in cancer patients (Kast, 1967). More recently, LSD has been shown to improve quality of life in patients with life-threatening disease (Gasser et al., 2014, 2015). Surveys have also described relief from cluster headache with LSD and psilocybin (Sewell et al., 2006; Schindler et al., 2015). Ayahuasca, the botanical brew containing DMT and a monoamine oxidase A inhibitor, produces an antidepressant effect and reduces symptoms of panic and hopelessness (Santos et al., 2007; Osório Fde et al., 2015; Sanches et al., 2016). There are ongoing studies investigating the effects of psychedelics in depression, drug and alcohol addiction, and headache disorders (Ross, 2012; Carhart-Harris et al., 2016). One of the most intriguing features of psychedelics’ therapeutic profile is the apparent persistence of therapeutic effects after limited exposure, such measures as antidepressant effects, cigarette smoking reduction/cessation (Grob et al., 2011; Gasser et al., 2015; Griffiths et al., 2016; Johnson et al., 2017a), and termination of cluster headache attacks (Sewell et al., 2006; Schindler et al., 2015). While the mechanisms of this ability to produce long-term effects are not fully understood, neuroplastic (Vollenweider and Kometer, 2010), genetic (Martin and Nichols, 2017), and psychological (Griffiths et al., 2008), processes are some of those postulated to be involved. The neuroendocrine system is another potential player in the lasting effects of psychedelics after limited exposure, particularly as the conditions shown to benefit from psychedelic therapy have demonstrable neuroendocrine aberrations. In this review, we describe certain structural and functional aspects of the neuroendocrine pathologies in neuropsychiatric disorders and cluster headache, as well as the effects that classic serotonergic psychedelics have on these systems. A summary of these descriptions can be found in Supplementary Table 1. Where applicable, those associations with the most supportive evidence for a persisting therapeutic effect will be discussed. This review will also serve to unify existing theories for the persisting effects of classic serotonergic psychedelics and highlight methodological strategies for future research in this area.

## Theories for Persisting Effects of Classic Serotonergic Psychedelics

### Pharmacology

Classic serotonergic psychedelics are those compounds that bind and activate the 5-HT2A receptor and cause significant alterations in sensorium and consciousness (Vollenweider et al., 1998; Nichols, 2004, 2016; Preller et al., 2017). While other drugs, such as 3,4-methylenedioxymethamphetamine (MDMA; ecstasy), Δ-9-tetrahydrocannabinol (Δ-9-THC), and ketamine, are often included in the category of psychedelic drugs and may have indirect effects on 5-HT2A receptors, their pharmacology is nevertheless distinct. For the purposes of this discussion, the pharmacologic definition of a 5-HT2A receptor agonist (or partial agonist) with psychotropic effects will be used when discussing psychedelics. The terms *psychedelic* and *hallucinogen* will also be used interchangeably.

The pharmacology of psychedelics has long been considered in their unique effects. The primary focus has involved the 5-HT2A receptor, as the binding affinity of psychedelics at this receptor is strongly correlated to the typical human dose for hallucinogenesis (Glennon et al., 1984; Sadzot et al., 1989). The roles of specific intracellular 5-HT2A receptor components and scaffolding proteins, such as ß-arrestin, have been considered in identifying a marker for hallucinogenesis (Schmid et al., 2008; Perez-Aguilar et al., 2014). The relative potencies and efficacies at activating 5-HT2A-mediated phosphatidylinositol (PI) hydrolysis and arachidonic acid (AA) release have also been investigated, but were not found to predict hallucinogenic potency or discriminate hallucinogenic from non-hallucinogenic drugs (Kurrasch-Orbaugh et al., 2003; Moya et al., 2007).

The density of 5-HT2A receptors can be manipulated to measure changes in the response to hallucinogens. For instance, repeated daily administration of the phenethylamine hallucinogen 2,5-dimethoxy-4-iodoamphetamine (DOI; 1.0 mg/kg i.p. daily × 8 days) in rats (McKenna et al., 1989) and rabbits (3 μmol/kg s.c. daily × 8 days) (Schindler et al., 2012) leads to a reduction in cortical 5-HT2A receptor density by about 50%. Serotonin2A receptor reduction is accompanied by significant attenuations in 5-HT-elicited PI hydrolysis signaling (Conn and Sanders-Bush, 1986; Ivins and Molinoff, 1991), as well as hallucinogen-elicited behaviors, such as head movements in rodents and rabbits (Leysen et al., 1989; Schindler et al., 2012; Moreno et al., 2013). In rats, chronic administration (daily for 8 days) of either LSD (60 μg/kg s.c.) or DOI (1.0 mg/kg s.c.) attenuated the locomotor inhibition induced by either drug (Krebs and Geyer, 1994). Similarly in rabbits, chronic administration of DOI (3 μmol/kg s.c. daily for 8 days) significantly decreased the head bob response to either DOI (3 μmol/kg s.c.) or LSD (30 nmol/kg s.c.) (Schindler et al., 2012). Such cross-tolerance was also shown in cats when a single dose of the hallucinogen 1-(2,5-dimethoxy-4-methylphenyl)-2-aminopropane (DOM; 4 mg/kg), LSD (0.2 mg/kg), or mescaline (50 mg/kg) attenuated DOM-elicited behaviors 24 h later (Wallach et al., 1974). In humans, tolerance, or tachyphylaxis, to a psychedelic’s effects occurs within about 3 days of daily exposure (Cholden et al., 1955; Belleville et al., 1956; Angrist et al., 1974); sensitivity returns in about as many days (Belleville et al., 1956). Unlike other psychedelics, however, DMT does not readily induce tolerance, which may be due to its short half-life or other yet unidentified factors (Strassman, 1996; Strassman et al., 1996). For instance, human subjects who received closely spaced repeated administrations (four doses at 30-min intervals) of intravenous DMT (0.3 mg/kg) failed to demonstrate tolerance to the psychedelic effects of the drug (Strassman et al., 1996). The ability of psychedelics to induce tolerance is relevant in the consideration of their use as therapeutic agents (i.e., identifying the appropriate intervals between doses).

The pharmacologic effects of limited or infrequent exposure to a psychedelic have not been extensively investigated, though they are sometimes reported in chronic administration studies. One group found that single administrations of LSD or DOI in rats did not affect cortical 5-HT2A receptor density at low doses (0.13 and 1.0 mg/kg i.p., respectively), but did so at high doses (0.65 and 7.0 mg/kg i.p., respectively) (Buckholtz et al., 1988, 1990). DOM reduced cortical 5-HT2A receptor density in rats after 2 doses (2.5 mg/kg s.c.) spaced 8 h apart (Leysen et al., 1989). In mice, a single dose of DOI (2.5 mg/kg i.p.) resulted in a significant *increase* in DOI-elicited head twitches out to 6 days, suggesting a super-sensitivity of the behavior (Darmani et al., 1992). Species differences are important to consider here. For one, genetic differences between mouse and human 5-HT2A receptor genes distinguish pharmacologic interactions with ligands (Canal et al., 2013). Furthermore, the binding properties of a number of serotonergic drugs in rabbits is more similar to those in humans than rats (Aloyo and Harvey, 2000). Additional studies examining the effects of single or intermittent (e.g., once weekly) dosing of psychedelics that include multiple measures (e.g., receptor density, behavior) taken at extended time points (e.g., out to a week or more) could help identify the pharmacologic substrate for persisting therapeutic effects.

In addition to the 5-HT2A receptor, psychedelics have appreciable activity at other serotonergic receptors, such as serotonin2C (5-HT2C) and serotonin1A (5-HT1A) receptors. The 5-HT2C receptor is involved in anxiety, dopaminergic neurotransmission, regulation of body weight, and addiction (Nichols and Nichols, 2008; Vengeliene et al., 2015; Canal and Murnane, 2017). Importantly, 5-HT2C receptors have been implicated in the lack of addictive properties of the hallucinogen drug class (Canal and Murnane, 2017). The serotonin1A receptor has been associated with neurogenesis, neuroprotection, depression, anxiety, dopaminergic neurotransmission, thermoregulation, and endocrine function (López et al., 1998; Nichols and Nichols, 2008). In animal studies, 5-HT1A receptor inhibition has been found to block various effects of psychedelics, such as drug stimulus cues (Winter et al., 2000; Fantegrossi et al., 2008) and locomotor activity reduction (Krebs-Thomson et al., 2006; Halberstadt et al., 2011). Across drugs, the importance of 5-HT1A receptor activation may differ, however (Nichols, 2016). For example, in rats, the drug stimulus cue of psilocybin was not affected by 5-HT1A receptor blockade (Winter et al., 2007), though the LSD cue was found to be modulated by 5-HT1A receptor activation (Reissig et al., 2005). In humans, 5-HT1A receptor blockade with pindolol (30 mg p.o.) enhanced the effects of a sub-hallucinogenic dose of DMT (0.1 mg/kg i.v.) in humans (Strassman, 1996). In addition to its 5-HT1A receptor inhibition, pindolol may enhance the effects of drugs through adrenergic inhibition (Schindler et al., 2013). The role of 5-HT1A receptor activation in neurogenesis has been associated with the therapeutic effects of antidepressants (Fricker et al., 2005; Samuels et al., 2015). In mice, a single low dose injection of psilocybin (0.1 mg/kg i.p.) tended to stimulate hippocampal neurogenesis 2 weeks after injection, though a high dose (1.0 mg/kg i.p.) inhibited it (Catlow et al., 2013). This dose effect may stem from counteractions mediated by 5-HT2A receptors (Klempin et al., 2010). Another receptor involved in hippocampal neurogenesis is sigma-1. Activation of sigma-1 receptors is similarly associated with a reduction in depressive behaviors in mice (Moriguchi et al., 2013, 2015). The sigma-1 receptor has also been associated with psychotropic drug effects (Su et al., 1988; Jansen et al., 1990; Ruscher et al., 2011). Ultimately, the actions at any one receptor cannot explain either the acute or persisting effects of these drugs. Additional systems associated with the action of psychedelics are dopaminergic, glutamatergic, and GABAergic systems (Vollenweider and Kometer, 2010; Nichols, 2016; Martin and Nichols, 2017).

### Genetics

Single doses of LSD and DOI induce a number of immediate early genes in various regions of rodent brain, including cortex, amygdala, nucleus accumbens, and striatum (Nichols and Sanders-Bush, 2002; Martin and Nichols, 2017). These various genes have been implicated in memory and synaptic plasticity and most remain active for several hours following drug treatment, which may initiate the processes involved with longer term phenotypic changes (Nichols and Sanders-Bush, 2002; Martin and Nichols, 2017). The induction of some genes, such as *c-fos* and *Arc*, is non-specific and seen with other serotonergic drug groups, such as antidepressants (González-Maeso et al., 2003; Gaska et al., 2012) and 5-HT2A receptor antagonist antipsychotics (Verma et al., 2006; Collins et al., 2014). The induction of *egr-1, egr-2*, and *period 1* genes was previously described as hallucinogen-specific as the effect was seen in mouse somatosensory cortex 1 h after LSD (0.24 mg/kg i.p.) and DOI (2 mg/kg i.p.) injection, but not lisuride (0.4 mg/kg i.p.) injection (González-Maeso et al., 2003). Gene induction likely depends on the model being used, however (Martin and Nichols, 2017). For example, *egr-2* expression was increased in rat cortical tissue cultures after LSD (10 μM), but not lisuride (10 μM), treatment (González-Maeso et al., 2007), though in a human study, LSD (100 μg p.o.) failed to alter expression of *egr-1, -2, or -3* in peripheral blood at 1.5 or 24 h after ingestion (Dolder et al., 2017). Thus, while gene activation studies offer a valuable means to identifying long-term effects, results should be interpreted with careful consideration.

### Epigenetics

Another possibility is that psychedelics may produce long lasting changes through epigenetic mechanisms. Decades ago, psychoactive doses of intravenously administered LSD were shown to rapidly increase histone acetylation in rabbit brain tissue (Brown and Liew, 1975). In contrast, another early experiment showed that neither LSD nor the phenethylamine hallucinogen, 3,4,5-trimethoxyphenethylamine (mescaline), inhibited interactions between nucleic acids and histone (Andersen et al., 1974). Although studies of the epigenetic effects of psychedelic drugs are extremely limited, future investigations may seek to focus on those components identified in related conditions. For instance, animal models of anxiety and depression have implicated methylation of the promoter in the serotonin transporter gene, *SLC64A*, and activity of histone deacetylase 6 (Holloway and Gonzalez-Maeso, 2015). Epigenetic modification of the glucocorticoid receptor gene, *NR3C1*, has also been associated with conditions of stress, (Moisiadis and Matthews, 2014b).

### Psychological Processes

The psychedelic experience itself has been suggested as a potentially beneficial or transformative therapeutic force with lasting effects. When administered under supportive conditions, psilocybin and LSD have been shown to result in peak experiences with substantial and sustained personal meaning and spiritual significance (Griffiths et al., 2006, 2008, 2011; Garcia-Romeu et al., 2014; Schmid and Liechti, 2017). Recent clinical trials of psychedelic drugs in the treatment of psychiatric disorders have demonstrated a correlation between the occurrence of such peak experiences and therapeutic benefits (Griffiths et al., 2011; Garcia-Romeu et al., 2014; Bogenschutz et al., 2015). The mechanisms by which peak experiences lead to these benefits are currently not well understood. If traumatic events are capable of causing epigenetic modifications within brain regions that influence behavior (Mathews and Janusek, 2011), as well as persistent structural and functional changes in limbic (Hull, 2002) and neuroendocrine systems (Najarian and Fairbanks, 1996; Yehuda et al., 1996; Raison and Miller, 2003) as observed in post-traumatic stress disorder (PTSD), then it is plausible that powerful positive or cathartic experiences, such as some psychedelic-occasioned peak experiences, “*may function as a salient, discrete event producing inverse PTSD-like effects – that is, persisting changes in behavior (and presumably the brain) associated with lasting benefit*” (Garcia-Romeu et al., 2014). While admittedly speculative, a powerful event holding significant salience could lead to epigenetic changes (Provencal et al., 2012; Black et al., 2013; Kaliman et al., 2014) or have effects on limbic circuitry that in turn alter neuroendocrine function, potentially reversing previously dysregulated systems caused by acute or chronic stress. This could help explain how psychedelic-assisted therapies not only have persisting effects, but why they may have therapeutic potential across a range of neuropsychiatric disorders.

Psychedelics have also been described as “*meaning-response magnifiers*” (Hartogsohn, 2016), serving to enhance the effects of placebo and set and setting. Indeed, LSD (40–80 μg i.v.) was found to enhance suggestibility in human subjects as measured by the creative imagery scale (Carhart-Harris et al., 2015). The subjective effects of LSD (2 μg/kg p.o.) have also been equated to those produced by hypnotic therapy, the combination resulting in more pronounced alterations in consciousness (Levine et al., 1963; Levine and Ludwig, 1965). The significance of such factors as intention, expectancy, preparation, and social setting in treatment outcomes is well recognized (Klosterhalfen and Enck, 2008; Hartogsohn, 2016). The placebo effect has also been discussed in the context of pain and reward circuitry (Klosterhalfen and Enck, 2008). A role for oxytocin has also been proposed (Enck and Klosterhalfen, 2009). As reviewed elsewhere (Zinberg, 1986; Eisner, 1997; Nichols, 2016), set and setting are well-known to influence the response to psychedelics. Studerus et al. (2012) studied the influence of several predictor variables on the acute response to psilocybin in pooled data from 23 controlled experimental studies involving 261 healthy volunteers who had participated in 409 psilocybin administrations. They confirmed that non-pharmacological factors play an important role in the effects of psilocybin (Studerus et al., 2012). Thus, high emotional excitability (set) and the experimental situation of undergoing positron emission tomography (PET) imaging (setting) most strongly predicted unpleasant and/or anxious reactions to psilocybin (Studerus et al., 2012). The interplay of psychedelics with a subject’s and the environment’s influence adds another facet to their potential therapeutic repertoire.

## Neuroendocrine Anatomy and Functional Imaging

The hypothalamus produces neuropeptides that regulate various biologic functions. The posterior hypothalamus, comprised of the paraventricular and supraoptic nuclei, produces oxytocin and vasopressin (or antidiuretic hormone), which are transported *via* the infundibulum to the posterior pituitary to be released into the blood. The anterior and lateral portions of the hypothalamus, comprised of several nuclei, produce such neuropeptides as corticotropin releasing hormone (CRH) and thyrotropin releasing hormone, which are released into the anterior pituitary to stimulate release of their respective hormones. Some such anterior pituitary hormones include adrenocorticotropic hormone (ACTH), thyroid stimulating hormone, prolactin, and orexin. Many biological functions are influenced by the neuroendocrine system and consequently, altered neuroendocrine function has association with a broad range of disorders.

The hypothalamus contains those receptors activated by psychedelics, including 5-HT2A (Appel et al., 1990; Zhang et al., 2004; Shi et al., 2008), 5-HT2C (Marazziti et al., 1999), 5-HT1A (Albert et al., 1990; Zhang et al., 2004; Moser et al., 2010; Dos Santos et al., 2015), dopamine (Mukherjee et al., 1999; Okubo et al., 1999), and sigma-1 (McLean and Weber, 1988) receptors (or mRNA). An early study demonstrated that acute injection of LSD (50 μg/kg i.p.) in rats increased “*neurosecretory materials*” in the excised posterior pituitary (Biswas and Ghosh, 1975). More recently, DOI (1 mg/kg s.c.) has been shown to induce serum increases of oxytocin, prolactin, ACTH, and corticosterone in rats, an effect blocked by either subcutaneous (Van de Kar et al., 2001) or intraparaventricular (blocked all except corticosterone) (Zhang et al., 2002) injection of 5-HT2A antagonist MDL100,907. Serotonin2A receptor binding in the paraventricular nucleus (PVN) of rats was decreased after repeated daily injections of DOI (1 mg/kg i.p. daily for 4 or 7 days), an effect accompanied by reduced DOI-induced serum oxytocin and ACTH levels (Shi et al., 2008). Interneurons and afferent fibers are likely to be involved with the neuroendocrine effects of psychedelics as well (Willins et al., 1997; Mackowiak et al., 1999; Van de Kar et al., 2001; Gresch et al., 2002). Indeed, cortical, subcortical, limbic, and brainstem inputs are involved with neuroendocrine regulation (Jorgensen, 2007; King and Liberzon, 2009). For example, serum cortisol increases in rhesus monkeys exposed to stress were associated with increased subgenual prefrontal cortex metabolism as measured by F-18-fluorodeoxyglucose (FDG) PET imaging (Jahn et al., 2010). In Vietnam combat veterans undergoing trauma recall, serum ACTH increases were associated with increased cerebral blood flow in the right insula and decreased activation of medial prefrontal cortex measured by [^15^O] H_2_O PET (King et al., 2009). In contrast, the so-called ACTH non-responders in this study activated medial prefrontal cortex and deactivated amygdala and hippocampus (King et al., 2009). Increased hypothalamic glucose metabolism has also been identified in depressed patients presented negative stimuli (Holsen et al., 2013; Im et al., 2016).

Functional brain imaging has shown that the inferior region of the posterior hypothalamus is activated during cluster attacks (May and Goadsby, 2001; Cohen and Goadsby, 2006). Cluster attacks are the paroxysms of cluster headache, a disorder characterized by episodes of unilateral retro-orbital pain so severe the disorder is coined “suicide headache” (Horton, 1952). In addition to activation, the volume of posterior hypothalamic gray matter is increased in cluster headache patients compared to healthy controls and appears slightly lateralized to the side of attacks (May et al., 1999). The posterior hypothalamus is also the target of deep brain stimulation (DBS) in the most refractory cases of cluster headache (Bartsch et al., 2009). It has been proposed that chronic stimulation of the posterior hypothalamus prevents activation, thus modulating activation of the trigeminal complex, resulting in pain relief (Leone et al., 2006; Bartsch et al., 2009). Indeed, after 1 month of posterior hypothalamic DBS activation in refractory cluster headache patients, sublingual nitroglycerin failed to trigger a cluster attack (*n* = 3) (Schoenen et al., 2005). Imaging has also served to identify pituitary lesions manifesting as a cluster headache syndrome, that improves or resolves with treatment of the particular lesion (Favier et al., 2007a,b).

Psychedelics produce measurable effects in the brain that may speak to their role in treating disease. In a review of neuroimaging studies, psychedelics are understood to generally increase prefrontal and limbic activity and decrease amygdala and default mode network activity, a combination that could serve to enhance interoception and cognition while blunting anxiety, fear, and rumination (Dos Santos et al., 2016). Vollenweider et al. (1997) reported that psilocybin (∼0.35 mg/kg p.o.) increased glucose metabolism in the brains of healthy human volunteers, increases in cortical regions being greater than those in subcortical regions (e.g., putamen). Similarly, in another human PET imaging study, psilocybin (0.2 mg/kg p.o.) increased the cortical/subcortical ratio of metabolism (on the right side) (Gouzoulis-Mayfrank et al., 1999a). This study specifically found *decreased* metabolism in subcortical regions relative to placebo (Gouzoulis-Mayfrank et al., 1999a). Another group found decreased amygdalar reactivity in healthy volunteers after oral psilocybin (0.16 mg/kg) ingestion (Kraehenmann et al., 2015). As measured by single photon emission tomography (SPECT), oral ayahuasca (2.2 mL/kg solution containing 0.8 mg/mL DMT) increased cerebral blood flow in the left nucleus accumbens, right insula, and left subgenual area, regions associated with mood regulation (Sanches et al., 2016). Intravenous LSD (75 μg i.v.) increased connectivity in frontal, parietal, and temporal cortices and bilateral thalami (Tagliazucchi et al., 2016). Taken together, these investigations may inform the neurobiological underpinnings of the therapeutic potential of psychedelics to treat depression, anxiety, and drug addiction (Dos Santos et al., 2016). One study specifically described decreased hypothalamic blood flow, as measured by arterial spin labeling and blood-oxygen level-dependent (BOLD) methods, after intravenous administration of psilocybin (2 mg) in healthy humans, which may hold relevance for treatment in cluster headache, although all brain regions of interest were found to have decreased blood flow in this particular study (Carhart-Harris et al., 2012).

Regarding cluster headache, it remains unknown how brief psychedelic exposure could affect the activation threshold of the hypothalamus or other relevant brain regions. The traditional dosing regimen for terminating cluster periods or inducing remission in chronic cluster headache is two to three doses, approximately 5 days apart, of psilocybin-containing mushrooms, LSD, or other psychedelics (Schindler et al., 2015; Andersson et al., 2017). How this traditional dosing regimen affects posterior hypothalamic anatomy and function is unknown, but could be investigated further with functional imaging, including a challenge of nitroglycerin (May et al., 1998) or another attack trigger, such as ethanol.

## Hypothalamus–Pituitary–Adrenal (HPA) Axis

In the well-described hypothalamus–pituitary–adrenal (HPA) axis, CRH from the anterior hypothalamus stimulates the release of ACTH from the anterior pituitary, which in turn acts in the adrenal gland to stimulate the release of such hormones as cortisol (corticosterone in rodents), aldosterone, and adrenaline (norepinephrine). With widespread actions, the HPA axis is best known for its roles in stress, metabolism, and inflammation (Silverman and Sternberg, 2012; Lemche et al., 2016). Manipulation of this system, even short-term, can have lasting effects. For instance, antenatal glucocorticoid exposure in humans has been associated with structural brain abnormalities, behavioral disturbances, and affective disorders from infancy to adulthood (Moisiadis and Matthews, 2014a). Childhood trauma (Lee et al., 2014) and repeated stressful life events in adulthood (Rutters et al., 2015) also increase the risk for metabolic syndrome. Epigenetic modification of the glucocorticoid receptor gene, *NR3C1*, has been documented in such conditions as maternal stress, childhood maltreatment, and war trauma (Ramo-Fernández et al., 2015). Moreover, these epigenetic, as well as behavioral and physiologic changes are reported to persist into subsequent generations (Moisiadis and Matthews, 2014b; Ramo-Fernández et al., 2015).

In otherwise healthy individuals with depressive symptoms, HPA axis abnormalities have also been identified, such as elevated basal cortisol levels (Halbreich et al., 1985) and abnormal responses to the dexamethasone suppression test (Carroll, 1982; Beck-Friis et al., 1985; Rubin et al., 1987), which normalize with treatment (Holsboer et al., 1982). Long-term exposure to prednisone, which mimics the biological effects of hypercortisolism in depression, is also associated with depressive symptoms (Patten and Barbui, 2004). In contrast to depression, individuals with PTSD show lowered baseline cortisol levels and greater cortisol suppression following a dexamethasone challenge (Najarian and Fairbanks, 1996; Yehuda et al., 1996; Raison and Miller, 2003). This is hypothesized to be secondary to the persistent intrusion of prior trauma leading to a repetition of the physiological stress response, thus altering (sensitizing) HPA functioning (Najarian and Fairbanks, 1996). In alcoholic patients, basal cortisol levels may vary depending on the amount of alcohol consumed (Boschloo et al., 2011). In abstinence, serum cortisol and serum and cerebrospinal fluid levels of ACTH did not differ among controls and alcoholics, though ACTH release induced by ovine CRH was suppressed in early abstinence (between 1 and 3 weeks) (Adinoff et al., 1990). In cluster headache, cortisol levels are increased during cluster periods, an effect that appears to be independent of headache pain or lack of sleep (Chazot et al., 1984; Leone and Bussone, 1993; Leone et al., 1995). Short term systemic glucocorticoid therapy is used in the treatment of cluster headache (Leone et al., 2017). There is also evidence for lasting effectiveness (weeks duration) after suboccipital steroid injection in cluster headache (Robbins et al., 2016; Leone et al., 2017).

Serotonin, as well as DOI, has been reported to stimulate CRH release from explanted rat hypothalami, containing the PVN, in a dose-dependent, inverted-U manner (Calogero et al., 1989). DOI and the related phenethylamine hallucinogen 1-(2,5-dimethoxy-4-bromophenyl)-2-aminopropane (DOB) both dose-dependently raised serum levels of ACTH and corticosterone in rats (Alper, 1990; Calogero et al., 1990; Owens et al., 1991; Hemrick-Luecke and Evans, 2002; Mikkelsen et al., 2004). ACTH and cortisol increases have also been found in humans after oral ingestion of LSD (Schmid et al., 2015a; Strajhar et al., 2016), psilocybin (Hasler et al., 2004), and ayahuasca (Dos Santos et al., 2012), as well as intravenous administration of DMT (Strassman and Qualls, 1994). Hormone increases are not specific to serotonergic psychedelics, however. Other psychotropic agents, such as MDMA (Gouzoulis-Mayfrank et al., 1999b; Seibert et al., 2014; Schmid et al., 2015b) and Δ-9-THC (Biswas and Ghosh, 1975; Mitra et al., 1977), also stimulate hormone production and release. Investigating functional outcomes (i.e., response to dexamethasone suppression) and epigenetic effects (i.e., *NR3C1*) after treatment may reveal additional therapeutic actions that are more specific to serotonergic psychedelics.

## Oxytocin

Oxytocin is a neuropeptide that plays a central role in social functions, particularly the attachment process, but also sexual behavior, maternal behavior, affiliation, and social memory (Insel, 1992; Insel, 1997; Van de Kar et al., 2001; Knobloch et al., 2012). Administration of oxytocin has anxiolytic and anti-depressive effects in rodents (Arletti and Bertolini, 1987; Neumann et al., 1999; Blume et al., 2008). While there have been mixed results about oxytocin levels in depression, certain oxytocin receptor single nucleotide polymorphisms (SNPs) have been associated with unipolar depression (Costa et al., 2009) and could be a mediator of selective serotonin reuptake inhibitor (SSRI) response (Uvnäs-Moberg et al., 1999). Oxytocin is also likely involved in the pathophysiology of PTSD and there is reason to believe it could be helpful in its treatment, particularly given its role in stress responsiveness, fear conditioning, and social functioning, all of which are impacted by PTSD (Van de Kar et al., 2001; Olff et al., 2010). Post-mortem examination of patients with alcohol disorder showed reduced oxytocin mRNA levels as compared to controls (Lee et al., 2017). In turn, intranasal oxytocin has been shown to reduce withdrawal symptoms in alcoholic patients (Pedersen et al., 2013). Oxytocin is further implicated in pain processing; oxytocin receptors are localized on trigeminal ganglion neurons, which directly implicates headache and facial pain disorders (Tzabazis et al., 2016). There is also support for therapeutic activity of oxytocin in migraine headache (Phillips et al., 2006; Serva et al., 2012; Tzabazis et al., 2016), which theoretically could extend to cluster and other headache types.

DOI (2.5 mg/kg i.p.) acutely increased oxytocin levels in rats, an effect shown to be 5-HT2A receptor mediated (Van de Kar et al., 2001). LSD (200 μg p.o.) also raised serum oxytocin levels in humans at 3 h (Schmid et al., 2015a). This stimulation of oxytocin by psychedelics could have implications for psychotherapy, as the administration of oxytocin during psychotherapy leads to changes in individual and dynamic factors in depressed patients (MacDonald et al., 2013) and in patients with PTSD (Koch et al., 2014). The proposed role of oxytocin in generating those elements required for placebo response (i.e., social interaction) supports the hormone’s potential function in a broad range of conditions (Enck and Klosterhalfen, 2009); cluster headache is included in this consideration, given the placebo effect of approximately 15% in prophylactic medication trials (Russell, 1979; Steiner et al., 1997; Leone et al., 2000; El Amrani et al., 2002; Hakim, 2011).

## Melatonin

Melatonin, a metabolite of serotonin, is produced in and secreted from the pineal gland, which receives modulatory input from the suprachiasmatic nucleus (SCN) of the anterior hypothalamus. Melatonin is secreted in times of darkness and has been extensively studied in circadian biology, serving as both a marker for and modulator of biologic rhythms (Raghavendra and Kulkarni, 2000; Lewy et al., 2006a). The role of melatonin in affective disorders has also been discussed in light of circadian disruption (Lewy, 2009). Serum melatonin levels and diurnal variation are aberrant in subjects with active depression and treatment with antidepressants modulate serum melatonin levels (Beck-Friis et al., 1985; Souetre et al., 1989; Srinivasan et al., 2006). A post-mortem study also showed reduced melatonin receptor 1 immunoreactivity in the SCN of depressed patients (Wu et al., 2013). In abstinent alcoholics, the nocturnal rise in melatonin was reported to be delayed (Kuhlwein et al., 2003). Melatonin levels are also low in cluster headache (Chazot et al., 1984; Leone et al., 1995), including times outside of cluster attack periods (Neeb et al., 2015), and the timing of melatonin release was found to be phase advanced (Chazot et al., 1984). Nightly melatonin (10 mg) has been shown to reduce the mean number of cluster attacks and terminate the cluster period in some patients (Leone et al., 1996). In addition, intravenous methylprednisolone (1,000 mg daily for 3 days) reduced cluster attack burden, while also raising aberrantly low levels of the melatonin metabolite, 6-sulfatoxymelatonin (Neeb et al., 2015).

*In vitro*, mescaline (1 μmol/L) and to a lesser extent, LSD (1 and 10 μmol/L) and psilocybin (10 and 100 μmol/L), stimulated melatonin release from rat pineal tissue, (Shein et al., 1971). *In vivo*, DOI (0.25–1.0 mg/kg i.p.) dose-dependently increased pineal melatonin content in rats, an effect blocked by pre-treatment with 5-HT2C antagonist, RS-102221 (2.5 mg/kg i.p.), but not 5-HT2A antagonist, ketanserin (6 mg/kg i.p.) (Steardo et al., 2000). In addition to serotonergic receptors (Govitrapong et al., 1991; Kaminski et al., 1993), dopaminergic (Kim et al., 2010; Gonzalez et al., 2012) and sigma-1 (Jansen et al., 1990) receptors (or mRNA) have been identified in the pineal gland. Psychedelics and melatonin have some opposing effects—psychedelics induce arterial hypertension, hyperthermia, anorexia, and HPA axis activation, whereas melatonin induces arterial hypotension, hypothermia, hyperphagia, and HPA axis suppression (Raghavendra and Kulkarni, 2000). Serving perhaps as a form of feedback, DOI (0.5 mg/kg i.p.) blocked melatonin-induced hypothermia, as well as serotonin release from the hypothalamus, in rats (Lin and Chuang, 2002). In turn, the suppression of food intake in rats induced by DOI (10 μg i.c.v.) was blocked by melatonin in a dose-dependent manner (1.5 and 3 mg/kg i.p.) (Raghavendra and Kulkarni, 2000). Understanding the normal rhythm of melatonin production and release is crucial for *in vivo* studies. For instance, intravenous DMT (0.4 mg/kg) did not acutely alter daytime serum melatonin levels in humans (Strassman and Qualls, 1994), but DOI (0.5 mg/kg i.p.) delayed the time of onset of urinary 6-sulfatoxymelatonin excretion by approximately 2.5 h in rats (Kennaway and Moyer, 1999). Furthermore, the delay in 6-sulfatoxymelatonin excretion induced by a single dose of DOI (0.5 mg/kg s.c.) was sustained for 8 days (Kennaway et al., 2001), illustrating the potential for long-term effects and the value of taking extended measures. Given that melatonin release was shown to be phase advanced in cluster headache (Chazot et al., 1984), this effect of DOI in rats may reveal part of mechanism by which psychedelics provide relief for patients with the disorder. In healthy human subjects, a single dose of the SSRI fluvoxamine (100 mg p.o.) also delayed melatonin release by approximately 2 h (Skene et al., 1994). The norepinephrine reuptake inhibitor, desipramine (100 mg p.o.), phase advanced melatonin release by 2–3 h, but it also increased 6-sulfatoxymelatonin excretion over a 48-h period (Skene et al., 1994). In another human study, the SSRI paroxetine (20 mg p.o.) and the anxiolytic (and 5-HT1A partial agonist) ipsapirone (20 mg p.o.) failed to alter serum melatonin levels over a 12-h period (Nathan et al., 1996). Antidepressants and anxiolytics are not effective in treating cluster headache and unlike psychedelics, single doses are not expected to have therapeutic effect. Be it melatonin or another hormone or marker, these studies do demonstrate that importance of collecting data at multiple time points for extended periods in order to best characterize the effects.

## Circadian Rhythm/Sleep

The SCN is the primary regulator of the circadian rhythm and receives afferent signals from retinal ganglion cells, highlighting the role of the environment (i.e., light) in the daily rhythm. The role of serotonin in SCN entrainment has also been described (Kronfeld-Schor and Einat, 2012). Disruption of the circadian rhythm through environmental stress, toxic exposures, or genetic mutation have been associated with various health repercussions (Masri and Sassone-Corsi, 2013; Perreau-Lenz and Spanagel, 2015). As an example, mice raised for the first 3 weeks of life in 24-h light conditions were shown to have increased CRH mRNA in the PVN and a depressive phenotype (Coleman et al., 2016). Maternal mouse exposure to a disrupted light-dark cycle led to signs of metabolic and affective abnormalities, as well as genetic changes, out to second and some third generation subjects (Zhang et al., 2017). In these second generation mice, a reduction in mRNA transcript levels of circadian clock genes (*CLOCK*, *BMAL1*, *PER1, PER2*) in the SCN were also identified (Zhang et al., 2017). Numerous animal studies have also shown that manipulation of clock genes results in behavioral and metabolic disturbances (Tsang et al., 2017). For instance, the manipulation of the clock genes, *CLOCK* and *PER2*, affected self-administration of addictive substances in rodents, though some gene associations are drug-specific (Perreau-Lenz and Spanagel, 2015). Affective and addictive conditions in humans have also been associated with clock gene SNPs (Partonen, 2015; Perreau-Lenz and Spanagel, 2015; Forde and Kalsi, 2017). The disrupted circadian rhythm is further supported clinically, as symptoms of depression show diurnal variation (Souetre et al., 1989) and sleep disturbance is common in depressed individuals (Tsuno et al., 2005) and those with alcohol use disorders (Kuhlwein et al., 2003; Brower, 2015).

The role of clock genes in cluster headache is also under investigation, though varying results are found (Russell, 2004; Fourier et al., 2017). Cluster headache is a particularly valuable model for studying biological rhythms. Circadian disruption, such as seasonal changes, shift work, and jet lag can trigger headache attacks (Chazot et al., 1984; Dodick et al., 2003). There is also a tendency for cluster periods to initiate or symptoms to worsen in spring and fall (Manzoni et al., 1983; Lund et al., 2017). Cluster attacks have the propensity to occur at predictable times of day as well (Manzoni et al., 1983; Lund et al., 2017), particularly during sleep and often during rapid eye movement (REM) sleep (Kudrow et al., 1984; Sahota and Dexter, 1990; Dodick et al., 2003). Interestingly, the polysomnogram of cluster headache patients (both inside or outside a cluster period) may show decreased number and frequency of REM sleep periods (Sahota and Dexter, 1990; Barloese et al., 2015), though REM sleep abnormalities are not always reported (Vetrugno et al., 2007). The alleviation of cluster headache symptoms after posterior hypothalamic DBS implantation may also be accompanied by changes in sleep quality and architecture, though these changes are not always pleasant (e.g., frequent overnight awakenings) (Vetrugno et al., 2007; Kovac et al., 2014).

In rats, LSD (1 mg/kg i.p.) postponed REM sleep onset (Depoortere and Loew, 1971). In cats, this delay of REM onset after LSD (25–800 μg/kg i.p.) was shown to occur in a dose-dependent manner (Brooks, 1975). Total REM sleep duration was also reduced after LSD in both rats (1 mg/kg i.p.) (Depoortere and Loew, 1971) and cats (2 μg/kg and 20 μg/kg LSD i.p.) (Hobson, 1964). This reduction in REM sleep duration after LSD (3.75, 7.5, 15 μg/kg i.v.) was shown to occur in a dose-dependent manner in cats (Kay and Martin, 1978). The non-hallucinogenic congener of LSD, 2-bromo-LSD (BOL; 3 mg/kg i.p.), also delayed REM sleep onset and reduced REM duration in rats (Depoortere and Loew, 1972), though to a lesser degree than LSD at the dose tested (Depoortere and Loew, 1971). In healthy humans, low doses of LSD (6–40 μg p.o.) given approximately at bedtime increased the duration of the first or second REM period, abbreviated subsequent REM periods, and induced REM bursts during slow wave sleep (Muzio et al., 1966). Another low dose of LSD (25 μg s.c.) administered in a healthy subject at bedtime advanced the first REM period and increased the ratio of REM to slow wave sleep (Toyoda, 1964). In one human subject under treatment for alcoholism, a high dose of LSD (300 μg p.o.) given mid-day led to a delay in the first REM period, an effect that persisted the following night (Green, 1965). Total duration of REM, isolated bursts of REM, gross body movements, and vocalizations, were *increased* in this patient the night of LSD exposure and the following two nights (Green, 1965). In another early study, sleep disturbances (grades of insomnia) were reduced for approximately 10 days after cancer patients took a single dose of LSD (100 μg, presumed to be oral) after breakfast (Kast, 1967). While it is not possible to generalize effects of LSD from this small number of subjects, the persisting effects are particularly noted. Furthermore, that psychedelics may delay the onset and reduce total duration of REM sleep (Hobson, 1964; Depoortere and Loew, 1971; Brooks, 1975; Kay and Martin, 1978) might suggest that one of their therapeutic benefits in cluster headache stems from manipulation of the sleep period during which attacks often occur. REM sleep duration may already be reduced in some cluster headache patients, however (Sahota and Dexter, 1990), and thus, psychedelics may not simply correct abnormal sleep patterns, but act on other related systems—through melatonin, for instance. In addition, REM sleep suppression is not unique to psychedelics; SSRI and tricyclic antidepressants, for instance, also acutely reduce REM sleep duration (Kantor et al., 2016; McCarthy et al., 2016). Distinctions in the effects of classic serotonergic psychedelics and other drugs may, again, be appreciated with longer-term monitoring of subjects.

In addition to taking repeated measures for an extended period, future studies examining sleep, circadian cycle, or other aspects of neuroendocrine function must also carefully consider timing of drug administration. For instance, melatonin administered at the end of the light phase, advanced the timing of peak water and ethanol drinking in alcohol-treated rats, but this shift was absent when melatonin was administered at the beginning of the light phase (Vengeliene et al., 2015). In humans, low doses of oral melatonin (0.225–0.3 mg/day) taken for 3 weeks led to decreased measures of depression in patients with seasonal affective disorder (SAD) when peak melatonin levels were achieved in the afternoon/evening as opposed to the morning (Lewy et al., 2006b). Given that most SAD patients are phase-delayed in their circadian rhythm, administering melatonin in the afternoon/evening (which causes phase advance) is conceptually favorable (Lewy et al., 2006a). Light therapy was also found to reduce depressed symptoms in SAD when administered in the morning (6–8am, 2500 lux, 1 week duration) as opposed to the evening (7–9pm) (Sack et al., 1990). Of note, this morning light therapy also advanced the onset of melatonin production (Sack et al., 1990). Time of day is also relevant in the consideration of neuroimaging studies. For example, between morning and evening, functional connectivity of the medial temporal lobe in humans was shown to expand to involve neocortical areas, suggesting a representation of memory consolidation (Shannon et al., 2013). In other human subjects, between morning and afternoon, default mode network connectivity decreased, an effect that also correlated with diurnal decreases in salivary cortisol levels (Hodkinson et al., 2014). In depressed patients, evening mood improvements were associated with increased metabolism in parietal and temporal cortices, basal ganglia, and the cerebellum, possibly reflecting a normalization required to preserve “*emotional homeostasis*” (Germain et al., 2007).

Given the desire to monitor subjects through the duration of psychotropic effects, studies investigating psychedelics in humans often administer drug early in the day. Though limited, early human studies showed that LSD produced differing effects on REM sleep, though doses and times of drug administration were quite variable (Toyoda, 1964; Green, 1965; Muzio et al., 1966; Kast, 1967). Animal models have further demonstrated the significance of the timing of administration of hallucinogenic compounds, however. For instance, disruption of the locomotor activity of house crickets was seen when LSD (5pg/g) was administered (injected into the hemolymph) early in the light phase, but not when administered late in the light phase (Cymborowski, 1970). In addition, LSD had no acute effects the day of injection, but reversed the locomotor rhythm of the house crickets the following day (Cymborowski, 1970). The hallucinogen 5-methoxy-*N,N*-dimethyltryptamine (5-MeO-DMT) (2–64 mg/kg i.p.) dose-dependently elicited head twitches in mice, an effect that was maximal in the middle of the light phase (Moser and Redfern, 1985). In contrast, another group reported that 5-MeO-DMT (5 mg/kg i.v.) elicited maximal head twitches in mice at the end of the dark period (Singleton and Marsden, 1981). In rats, DOI dose-dependently induced wet dog shakes, a response that peaked late in the light phase after either subcutaneous or intracerebroventricular injection (0.5 mg/kg) (Nagayama and Lu, 1996). In addition to differences among species and routes of administration, the methods of measuring time points must be considered in the effects of psychedelics. For instance, as discussed previously, a single subject dosed multiple times may develop tolerance to a drug, whereas different subjects each dosed at a time point of interest would better reflect the effects of a single administration.

## Discriminating the Effects of Psychedelics

Psychedelics are best known for their ability to alter one’s consciousness, which has afforded them both fame and infamy. There are actions of classic serotonergic psychedelics unrelated to hallucinogenesis, however. Indeed, various systemic targets of psychedelics, such as heart rate and blood pressure, are commonly measured alongside psychotropic effects (Strassman and Qualls, 1994; Hasler et al., 2004; Schmid et al., 2015a). Anti-inflammatory and anti-cancer effects of psychedelics have also been described (Szabo, 2015). Regarding the topic of this report, psychedelic drugs target the anatomical and biochemical substrates of neuroendocrine function. Both central and peripheral actions are involved. For example, psychedelic-induced increases in corticosterone have been shown to involve both peripheral (i.e., direct adrenal) and central (i.e., ACTH-mediated) mechanisms (Alper, 1990; Calogero et al., 1990; Owens et al., 1991). While the peak psychotropic effects of oral LSD (200 μg) (Schmid et al., 2015a; Strajhar et al., 2016), psilocybin (315 μg/kg) (Hasler et al., 2004), and ayahuasca (oral DMT portion 0.75 mg/kg) (Dos Santos et al., 2012) in humans approximately coincide with maximal serum hormone increases, a separation between these measures can also be shown. For instance, low, though still psychoactive, doses of psilocybin—from 45 μg/kg (p.o) (Hasler et al., 2004) to 200 μg/kg (p.o.) (Gouzoulis-Mayfrank et al., 1999b)—did not significantly change the levels of various hormones, including ACTH, cortisol, prolactin, thyroid stimulating hormone, and growth hormone, at multiple time points out to 300 min. Furthermore, when administered intravenously, DMT (0.2 and 0.4 mg/kg)-induced psychological effects peaked at 5 min, the approximate time of peak ACTH and prolactin elevation (5–10 min), but well preceding maximum cortisol levels (15–30 min) (Strassman and Qualls, 1994). Four closely spaced (30-min intervals) doses of intravenous DMT (0.3 mg/kg) in humans led to tolerance of ACTH, cortisol, and prolactin stimulation, but not the psychedelic effects of the drug, (Strassman et al., 1996). This separation between psychotropic and endocrine effects underscores the multiple actions of psychedelics.

The delayed and/or sustained effects on sleep and melatonin measured in both human (Green, 1965; Muzio et al., 1966; Kast, 1967) and non-human (Kennaway and Moyer, 1999; Kennaway et al., 2001) animals are also examples of the separation between psychotropic and other effects. That BOL, as well as low doses of LSD, can affect sleep architecture in a similar manner to psychoactive LSD doses lends further support to actions independent of psychotropic effects (Muzio et al., 1966; Depoortere and Loew, 1972). To be precise, oral BOL ingestion in humans does not induce psychedelic effects (Richards et al., 1958), although “flabby” or “light drunk” feelings have been described (Karst et al., 2010). In one early case report, BOL (0.5 mg p.o.) induced sensory perceptual changes, panic, and cardiovascular and gastrointestinal activation in one subject (Richards et al., 1958). The source and purity of BOL in this case was not identified, however. Of note, the subject in this early report had ingested BOL after the development of a pounding headache, which was reduced in intensity from moderate to mild (Richards et al., 1958). Anecdotally, patients have reported lasting relief from cluster headache after ingesting BOL (Schindler et al., 2015). In a case series, BOL (30 μg/kg p.o.) was shown to reduce cluster attack burden in the same 3-dose regimen as for hallucinogenic psychedelics (Karst et al., 2010). While the pharmacologic effects of BOL have not been fully examined, the general consensus that it has greatly reduced (or no) hallucinogenic properties, raises the question as to the necessity of psychotropic effects in treatment with classic serotonergic psychedelics. Indeed, sub-hallucinogenic doses of psilocybin and LSD are also reported to provide relief from cluster headache in some patients (Sewell et al., 2006; Schindler et al., 2015). There are widespread anecdotal reports of sub-hallucinogenic doses of psychedelics being beneficial in a range of psychiatric illnesses *via* so-called “*micro-dosing*” protocols as well, though clinical trials are lacking (Fadiman, 2011). The persisting effects of psychedelics in cluster headache may be independent in origin from those in neuropsychiatric disorders.

## Conclusion

There is ongoing interest in the study of classical serotonergic psychedelics in the fields of pharmacology, epi/genetics, neuroimaging, and psychology. The neuroendocrine system should be considered among the many potential targets for lasting therapeutic benefit. In mood and substance use disorders, HPA axis function is widely studied. The manipulation of this system can have demonstrable long-term effects and should be of interest in considering the additional non-psychological effects of psychedelics in the treatment of neuropsychiatric disease. In cluster headache, aberrations in melatonin and circadian rhythm are topics of value in examining the effects of psychedelics. With advancing understanding of circadian biology (e.g., clock genes), psychedelics should be actively considered in this process. Importantly, given the associations with the neuroendocrine system, future studies examining the effects of psychedelics must take into account the timing and pattern of drug administration, as well as frequency and duration of outcome measures. Finally, though incomplete, existing evidence raises the intriguing possibility that as a class, psychedelics could have therapeutic effects independent from their hallucinogenic effects. Pharmacologically similar, but non-hallucinogenic compounds, such as BOL, should also be utilized in examining the role of hallucinogenesis in the therapeutic effects of this drug class.

## Author Contributions

ES is the primary author. She conceived the topic and the design of the manuscript and drafted and critically revised the manuscript. RW is a significant contributing author. He provided substantial contributions to the design and content of the manuscript and critically revised the manuscript. JS is a significant contributing author. He provided substantial contributions to the design and content of the manuscript and critically revised the manuscript. DD is the last author. He provided substantial contributions to the design and content of the manuscript and critically revised the manuscript. All authors approved the final version of the manuscript and agreed to be accountable for all aspects of the work.

## Conflict of Interest Statement

The authors declare that the research was conducted in the absence of any commercial or financial relationships that could be construed as a potential conflict of interest.
