# Supplementary material for: Neuroendocrine Associations Underlying the Persistent Therapeutic Effects of Classic Serotonergic Psychedelics
Source: Front Pharmacol. 2018 Mar 1;9:177. doi: 10.3389/fphar.2018.00177 (PMC5838010; doi:10.3389/fphar.2018.00177)
Supplement: Supplementary file 1 [file Table_1.PDF]

Table 1: Summary of neuroendocrine associations

System	Role in Affective / Substance Use Disorders	Role in Cluster Headache	Effect of Psychedelics
Hypothalamus	-increased hypothalamic glucose metabolism in depressed patients presented negative stimuli (Holsen et al., 2013;Im et al., 2016)<br><br>-mice raised in 24-hour light (stress) had increased CRH mRNA in the PVN and depressive phenotype (Coleman et al., 2016)	-activation of posterior hypothalamus in cluster headache (May and Goadsby, 2001;Cohen and Goadsby, 2006)<br><br>-increased gray matter lateralized to side of attacks (May et al., 1999)<br><br>-DBS in posterior hypothalamus used to treat cluster headache (Schoenen et al., 2005;Bartsch et al., 2009)	-DOI induced CRH release from explanted rat hypothalamus (Calogero et al., 1989)<br><br>-DOI-induced serum increases of oxytocin, prolactin, ACTH in rats blocked by intraparaventricular 5-HT2A antagonist (Zhang et al., 2002)<br><br>-Decreased hypothalamic blood flow after intravenous psilocybin in humans (Carhart-Harris et al., 2012)
Pituitary gland	--	-Pituitary lesions can produce cluster phenotype (Favier et al., 2007a;Favier et al., 2007b)	-LSD stimulated *"neurosecretory materials"* in the posterior pituitary of rats (Biswas and Ghosh, 1975)
Pineal gland	--	--	-Mescaline applied to pineal tissue induced melatonin release; nonsignificant increases with LSD, psilocybin (Shein et al., 1971)
Hormones			
HPA Axis (e.g. ACTH, cortisol)	-elevated baseline cortisol and abnormal response to dexamethasone suppression test in depression (Carroll, 1982;Halbreich et al., 1985;Rubin et al., 1987)<br><br>-decreased cortisol and heightened response to dexamethasone suppression test in PTSD (Najarian and Fairbanks, 1996;Yehuda et al., 1996;Raison and Miller, 2003)<br><br>-chronic steroid treatment leads to depressed phenotype (Patten and Barbui, 2004)<br><br>-maternal stress and other trauma can lead to modification of *NR3C1* glucocorticoid receptor that persists into subsequent generations (Moisiadis and Matthews, 2014;Ramo-Fernandez et al., 2015)	-increased baseline cortisol in cluster headache (Chazot et al., 1984;Leone and Bussone, 1993;Leone et al., 1995)<br><br>-glucocorticoid pulse used in treatment of cluster headache (Neeb et al., 2015;Leone et al., 2017)	-DOI and DOB raised serum ACTH and corticosterone in rats (Alper, 1990;Calogero et al., 1990;Owens et al., 1991;Hemrick-Luecke and Evans, 2002;Mikkelsen et al., 2004;Shi et al., 2008)<br><br>-LSD (Schmid et al., 2015;Strajhar et al., 2016), psilocybin (Hasler et al., 2004), ayahuasca (Dos Santos et al., 2012), and DMT (Strassman and Qualls, 1994) increased serum cortisol and ACTH in humans

	-ACTH induction blunted in early alcohol abstinence (Adinoff et al., 1990)		
Oxytocin	-anxiolytic and antidepressant effects in rats (Arletti and Bertolini, 1987;Neumann et al., 1999;Blume et al., 2008)<br><br>-oxytocin receptor SNPs associated with depression (Costa et al., 2009) and SSRI response (Uvnäs-Moberg et al., 1999)<br><br>-oxytocin reduced depressed symptoms in SAD (Lewy et al., 2006)<br><br>-oxytocin peptide mRNA reduced in prefrontal cortex of humans with alcohol use disorder (post-mortem) (Lee et al., 2017)<br><br>-intranasal administration reduced symptoms of alcohol withdrawal in humans (Pedersen et al., 2013)	-oxytocin receptors on trigeminal ganglion neurons (Tzabazis et al., 2016)<br><br>-oxytocin is a potential therapy in migraine (Phillips et al., 2006;Serva et al., 2012;Tzabazis et al., 2016)	-DOI increased serum levels in rats (Van de Kar et al., 2001)<br><br>-LSD increased serum levels in humans (Schmid et al., 2015)<br><br>-role for oxytocin in suggestibility and placebo response (Enck and Klosterhalfen, 2009)
Melatonin	-abnormal levels in depression (Srinivasan et al., 2006)<br><br>-reduced melatonin receptor 1 immunoreactivity in the suprachiasmatic nucleus of depressed patients (Wu et al., 2013)<br><br>-nocturnal rise delayed in abstinent alcoholics (Kuhlwein et al., 2003)	-low levels in cluster headache (Chazot et al., 1984;Leone et al., 1995;Neeb et al., 2015)<br><br>-phase advanced release in cluster headache (Chazot et al., 1984)<br><br>-nightly melatonin reduced cluster headache burden (Leone et al., 1996)	-Mescaline applied to pineal tissue induced melatonin release; nonsignificant increases with LSD, psilocybin (Shein et al., 1971)
Circadian/Sleep	-sleep disturbance common in affective and addictive disorders (Kuhlwein et al., 2003;Tsuno et al., 2005;Brower, 2015)<br><br>-circadian nature of depressive symptoms (Souetre et al., 1989)<br><br>-maternal stress led to reduction in clock gene mRNA in SCN out to the 2nd generation of	-circadian nature of cluster headache (time of year and time of day) (Manzoni et al., 1983;Lund et al., 2017)<br><br>-circadian disruption triggers cluster attacks (Chazot et al., 1984;Dodick et al., 2003)	-LSD postponed REM onset and reduced total REM in rats (Depoortere and Loew, 1971) and cats (Hobson, 1964)<br><br>-LSD advanced the first REM period, increased REM to slow wave sleep ratio (Toyoda, 1964)<br><br>-LSD altered REM pattern in healthy humans (Muzio et al., 1966)

	offspring (Zhang et al., 2017)<br><br>-clock gene SNPs identified in affective and addictive conditions (Partonen, 2015;Perreau-Lenz and Spanagel, 2015;Forde and Kalsi, 2017)<br><br>-manipulation of clock genes altered drug self-administration in rodents (Perreau-Lenz and Spanagel, 2015)	-cluster attacks occur often during REM sleep (Kudrow et al., 1984;Sahota and Dexter, 1990;Dodick et al., 2003)<br><br>-patients may have decreased total REM duration (Sahota and Dexter, 1990;Barloese et al., 2015)	-LSD delayed REM onset and increased REM duration for up to three nights after one large dose in an alcoholic patient (Green, 1965)<br><br>-LSD improved subjective sleep quality in cancer patients for 10 days after a single moderate dose (Kast, 1967)
